# GATA6 inhibits the biological function of non-small cell lung cancer by modulating glucose metabolism

**DOI:** 10.1007/s00432-024-05664-y

**Published:** 2024-03-14

**Authors:** Weiwei Ju, Lijuan Lin, Qifang Zhang, Xiumei Lv, Shaohui Teng, Yu Hong, Zhixiang Shao, Hanyun Na, Shengjin Yu

**Affiliations:** 1 Institute of Molecular Medicine, Medical College of Liaodong University, Dandong, 118003 China; 2 Pathology Department, Dandong First Hospital, Dandong, 118003 China

**Keywords:** GATA6, Non-small cell lung cancer, Glucose metabolism, c-Myc

## Abstract

**Purpose:**

This study aims to explore the role of GATA6 in lung cancer, with a focus on its impact on metabolic processes.

**Methods:**

We assessed GATA6 expression in lung cancer tissues and its association with patient prognosis. In vitro cell function experiments were conducted to investigate the effects of altered GATA6 levels on lung cancer cell proliferation and migration. Mechanistic insights were gained by examining GATA6's influence on glucose metabolism-related genes, particularly its effect on c-Myc mRNA expression.

**Results:**

Our study revealed significant down-regulation of GATA6 in lung cancer tissues, and this down-regulation was strongly correlated with unfavorable patient prognosis. Elevating GATA6 levels effectively inhibited the proliferation and migration of lung cancer cells in our cell function experiments. Mechanistically, we found that GATA6 suppressed the expression of c-Myc mRNA, impacting genes related to glucose metabolism. As a result, glucose uptake and metabolism in lung cancer cells were disrupted, ultimately impeding their malignant behaviors.

**Conclusion:**

Our study provides crucial insights into the metabolic regulation of GATA6 in lung cancer cells. These findings have the potential to offer a solid theoretical foundation for the development of novel clinical treatments for lung cancer.

## Introduction

Lung cancer ranks among the most prevalent and lethal malignancies globally (Mountzios et al. 2023). With an overall five-year survival rate of less than 20%, lung cancer lags behind many other major cancer types in terms of prognosis (Osarogiagbon et al. 2023). Non-small cell lung cancer (NSCLC) constitutes the primary subtype, accounting for approximately 85% of lung cancer cases, with a staggering 84% of NSCLC diagnoses occurring in advanced stages (Howard and Pearson 2020; Chen et al. 2023). Consequently, the early detection and timely intervention of lung cancer are paramount for improving patient survival rates. Identifying prognostic protein molecules is, thus, of utmost importance.

GATA6, a member of the GATA transcription factor family (GATA1-6), plays a pivotal role in embryonic growth and differentiation (Tremblay et al. 2018; Romano and Miccio 2020; Dobrzycki et al. 2020; Moriguchi 2021). Numerous studies have indicated a close association between GATA6 expression levels and the development of NSCLC. Retinoic acid (RA) has been shown to mitigate tyrosine kinase inhibitor (TKI) resistance by upregulating GATA6 expression, consequently suppressing EGFR transcription and inhibiting Wnt signaling activation (Zito et al. 2017). Conversely, another study has demonstrated that elevating GATA6 levels enhances autophagy, fostering the progression of chemotherapy resistance in lung cancer cells (Ma et al. 2019). Furthermore, miR-196b has been found to promote the migration and invasion of lung cancer cells by targeting GATA6 levels (Liang et al. 2020; Li et al. 2018), while miR-200 downregulates bone morphogenetic protein-4 (BMP4) expression by directly reducing GATA6 gene transcription, thereby impeding lung cancer cell growth and metastasis (Yu et al. 2022). These findings underscore the intricate relationship between GATA6 and NSCLC, highlighting the need for comprehensive research to fully elucidate the role of the GATA6 gene and provide more precise targets for NSCLC diagnosis and treatment.

In our study, we observed down-regulation of GATA6 expression in lung cancer tissues, with tumors exhibiting lower GATA6 expression levels showing a propensity for larger tumor volumes and lymph node metastasis. Furthermore, GATA6 expression in lung cancer tissues correlated with patient prognosis. Our cell function experiments also demonstrated that elevated GATA6 expression effectively curtailed the proliferation and migration capabilities of lung cancer cells. Mechanistically, we uncovered that GATA6 influences the expression of genes associated with glucose metabolism by inhibiting c-Myc mRNA expression. This inhibition subsequently curtailed glucose uptake and metabolism by lung cancer cells, ultimately hindering their malignant biological behaviors. Our study thus provides detailed insights into the specific mechanisms through which GATA6 regulates lung cancer cell functions from a metabolic perspective, offering a robust theoretical foundation for clinical lung cancer treatment.

## Materials and methods

### Patients and tissue samples

Tissue specimens of patients with NSCLC undergoing surgical resection in Dandong First Hospital from January 2010 to December 2013 were collected. The inclusion criteria were as follows: (1) None of the patients had received radiotherapy, chemotherapy or other treatment before surgery; (2) The tissue specimen was confirmed as lung adenocarcinoma by histopathology. A total of 150 patients were collected, ranging in age from 38 to 86 years, with a median age of 60 years. A total of 101 cases were followed up with complete information. This study was approved by the Ethics Committee of Dandong First Hospital, and all patients signed informed consent.

### Immunohistochemical (IHC) staining and scoring

Antibodies GATA6 (1:200, ab175349, abcam), c-Myc (1:200, ab32072, abcam), Ki67 (1:1000, ab15580, abcam) and Cleaved Caspase-3 (1:200, 9961, CST) were used. The first antibody diluent for immunohistochemistry, the second antibody labeled with horseradish peroxidase (PV-6000) and the DAB color development kit were all purchased from Beijing Zhongshan Jinqiao Biotechnology Co., LTD. SP two-step method was used for staining. The primary antibodies were incubated at 4 °C overnight, and the secondary antibody was incubated at room temperature for 1 h, then the color was developed by DAB for 2 min. For the immunohistochemical results of GATA6, the number of positive cells and positive intensity were comprehensively considered, and a semi-quantitative method was adopted to score 0 points (0), 1 points (1–25%), 2 points (26–50%), 3 points (51–75%), and 4 points (76–100%) according to the proportion of positive cells, and 0 (no staining), 1 (lower staining), 2 (moderate staining), and 3 (higher staining) according to the coloring intensity. The two scores were multiplied, and 10 high-power field scores were randomly selected for each section in the hot spot area. Finally, all cases were divided into two groups according to the score results, 0–4 was divided into low-expression group and 5–12 was high-expression group. Immunohistochemical results of c-Myc, Ki67 and Cleaved Caspase-3 were analyzed statistically according to the percentage of positive cells.

### Cell culture and cell line construction

Human lung adenocarcinoma cell lines A549 and PC9 were obtained from the ATCC, and cultured in medium 1640 containing 10% fetal bovine serum at 37 °C in a constant temperature incubator with 5% CO_2_ volume fraction and saturated humidity. We confirmed the authenticity of all cell lines through short tandem repeat profiling and conducted tests to ensure the absence of *Mycoplasma* contamination. The plasmids and lentiviruses with overexpression or down-expression of GATA6, as well as the control plasmids and viruses were synthesized and packaged by Gene-Chem (Shanghai Genechem Co., Ltd.). The lung cancer cells were planted on the six-well plate, and the amount of virus required was calculated according to the number of infections to infect the cells of logarithmic growth stage. After 48 h, cells were screened with 1 mg/L purinamycin for 2 days. Then, the expression level of GATA6 was detected by real-time PCR and western blot to judge the infection effect.

### RNA extraction and real-time PCR

Total cell RNA was extracted using TRIzol reagents (Invitrogen), quantified using NanoDrop 1000 (Thermo Fisher), and its quality was evaluated by gel electrophoresis. Real-time PCR was performed using Power SYBR Green (TaKaRa Company) with GAPDH as the internal control. The primers were: GATA6 upstream primer 5′-TGCAATGCTTGTGGACTCTA-3′, downstream primer 5′-GTGGGGGAAGTATTTTTGCT-3′; GAPDH upstream primer 5′-AAGGTGAAGGTCGGAGTCAA-3′, downstream primer 5′-AATGAAGGGGTCATTGATGG-3′.

### Western blot

Protein was extracted from RIPA lysate (Solebao), and protein concentration was measured and homogenized by BCA method. 10% SDS-PAGE gel electrophoresis was performed for 90 min, and the protein was wet-transferred on PVDF membrane by Bio-Rad device for 250 mA for 2 h. The PVDF film containing the target strip was cut and sealed with 5% milk for 1 h at room temperature. GATA6 antibody (1:1000), c-myc antibody (1:1000), GAPDH (Santa Cruz Corporation, 1:5000) and β-actin (Santa Cruz Corporation, 1:5000) were incubated overnight at 4 °C. Incubation of the secondary antibody (Cell Signaling Technology, 1:10,000) at room temperature for 1 h and ECL development (millipore).The Image J software was employed for the quantification of protein band intensities, with the ratio between the gray value of the target protein band and that of the internal reference protein band serving as a metric for assessing the relative expression of the target protein.

### CCK-8 cell proliferation assay

The cells with logarithmic growth were inoculated into 96-well plates (5 × 10^3^ cells /200 μL) with five replicates in each group. At 24-, 48-, 72-, 96-, and 120-h time points, respectively, the original culture medium was discarded and 100 μL culture medium containing 10% CCK-8 was added. After incubation for 2 h, OD values of each hole were measured at 450 nm, and growth curves were drawn.

### Two-dimensional clonal formation assay

Cells in the logarithmic growth phase were seeded into six-well plates at a density of 1000 cells per well and incubated for a period ranging from 10 to 14 days. Upon the emergence of visible colonies, the supernatant was aspirated, and the clones were fixed by adding methanol at −20 °C for 6 min. Subsequently, a 0.1% crystal violet solution was applied at room temperature for 30 min, and the number of colonies containing more than 50 cells was enumerated using an optical microscope.

### Transwell assay

The cell lines at logarithmic growth stage were taken for the preparation of single-cell suspension. After centrifugation, 10,000 cells were added to the upper chamber of the cell, and 500 μl of 10% FBS was added to the 24-well plate, and the cell was placed in the 24-well plate. After 24 h of culture, the cells in the upper chamber that did not cross the compartment membrane were wiped with cotton swabs. After crystal violet staining, the images were collected under the microscope. Dozens of visual fields were randomly counted under a 40-fold optical microscope for statistical analysis.

### Isotope tracer analysis of ^13^C-labeled glucose

A549 cells were subjected to a 12-h incubation with a culture medium containing 10 mM [U-13C6] glucose. After removing the medium, metabolites were extracted by adding 80% cold methanol. The resulting lysate was preserved at −80 °C for 2 h, followed by centrifugation at 4 °C at 12,000*g* for 20 min to eliminate macromolecules and debris. The supernatant was subsequently lyophilized under a nitrogen stream at low temperature to obtain a powdered form of the metabolites. These metabolites were re-suspended in 50 μL of 80% methanol, with 1 μL per sample injected into a 2.1 × 100 mm Synergi hydrop-rp 100A column maintained at 35 °C (Phenomenex, Torrance, CA, USA). Data obtained from multiple reaction monitoring were analyzed using Tracefinder software (Thermo Fisher Scientific) for metabolite quantification in flux analysis. Finally, relative metabolite abundances between samples were compared.

### Analysis of luciferase activity

Using the Promega Dual-Luciferase™ Reporter Gene (DLR™) assay system according to the instructions, the c-Myc reporter plasmid and control plasmid were constructed by Gene-Chem (Shanghai Genechem Co., Ltd). Cells were inoculated into 96-well plates at a density of 2 × 10^3^ cells per well, and corresponding plasmids were transfected. After 24 h, the cells were lysed and the activity of firefly and kidney cells was measured. The luciferase signal was normalized with renilla values.

### Public database

The expression level of GATA6 mRNA in lung adenocarcinoma and normal lung tissues was analyzed by interactive gene expression profile analysis of TCGA database (GEPIA, http://gepia.cancer-pku.cn/). Using KMPLOT database (http://kmplot.com/analysis/) analysis GATA6 mRNA on the survival of patients with lung adenocarcinoma.

### Statistical methods

All experimental data were subjected to statistical analysis using SPSS 16.0 software. The χ^2^ test was employed to assess the correlation between the expression level of GATA6 protein and the clinicopathological indicators of the patients. Kaplan–Meier univariate survival analysis was conducted to investigate the association between the expression level of GATA6 protein and the patients' survival prognosis. In cell experiments, a *t*-test was utilized for statistical analysis to compare differences between the experimental and control groups. A significance level of *P* < 0.05 was considered statistically significant in all analyses. Each experiment was independently repeated more than three times to ensure robustness and reliability of the results.

## Results

### The expression of GATA6 in NSCLC tissues and its association with clinicopathological features

**Fig. 1:**
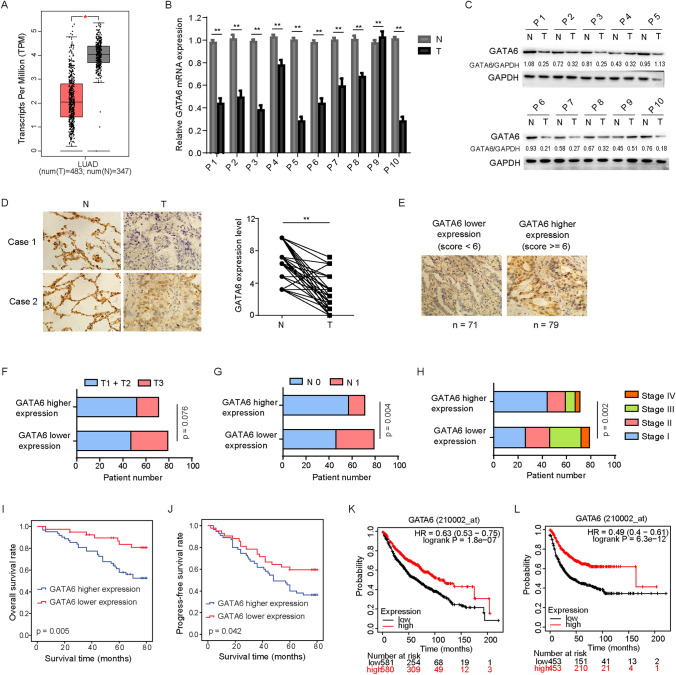
The expression of GATA6 in lung cancer and its relationship with clinicopathological indexes. **A** Expression level of GATA6 mRNA in lung adenocarcinoma and normal lung tissues in TCGA database. **B** GATA6 mRNA expression levels in NSCLC tissues and adjacent lung tissues. **C** Expression level of GATA6 protein in NSCLC tissues and adjacent lung tissues was detected by western blot. **D** The expression level of GATA6 protein in NSCLC and adjacent lung tissues was detected by IHC. **E** IHC representation of high- and low-expression GATA6 protein. **F**–**H** Relationship between the expression level of GATA6 protein and T stage (**F**), N stage (**G**) and TNM stage (**H**) in lung adenocarcinoma. **I**, **J** The association between GATA6 protein expression in NSCLC and both overall patient survival (**I**) and progression-free survival (**J**). **K**, **L** The association between GATA6 protein expression in lung adenocarcinoma and both overall patient survival (**K**) and progression-free survival (**L**) in Kmplot database

**Fig. 2:**
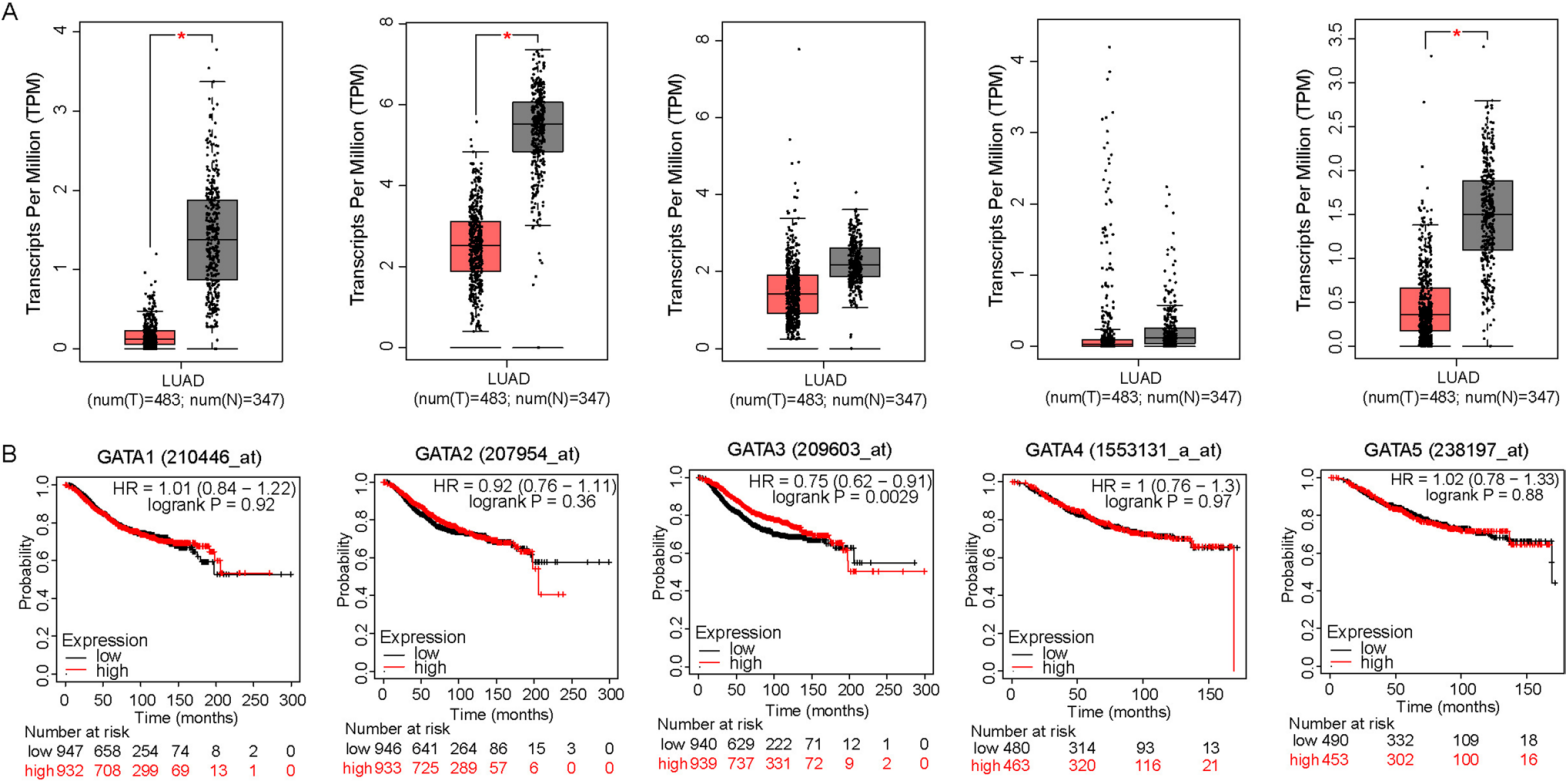
Expression of GATA family members in lung cancer and its relationship with patient prognosis. **A** mRNA levels of GATA family members in lung adenocarcinoma and normal lung tissues in TCGA database. **B** Relationship between mRNA levels of GATA family members in lung adenocarcinoma and overall survival of patients in Kmplot database

To delineate the expression pattern of GATA6, we initially conducted an analysis of GATA6 mRNA expression levels in lung adenocarcinoma and normal lung tissues, utilizing data sourced from the TCGA database. Our findings unequivocally demonstrated that GATA6 mRNA expression in lung adenocarcinoma tissues was markedly diminished compared to that in normal lung tissues (Fig. 1A). Subsequently, we procured 10 pairs of fresh NSCLC tissues, along with their corresponding adjacent normal lung tissues, revealing a consistent reduction in both mRNA levels (Fig. 1B) and protein levels (Fig. 1C) of GATA6 within NSCLC specimens relative to normal lung tissues. Furthermore, immunohistochemistry (IHC) analyses performed on 150 patients corroborated these observations, manifesting a pronounced decrease in GATA6 expression in NSCLC tissues (Fig. 1D). Based on these IHC results, we categorized the 150 cases into two groups: the GATA6 low-expression group and the GATA6 high-expression group (Fig. 1E). Intriguingly, as shown in Table 1, we established a significant negative correlation between GATA6 expression levels and crucial clinicopathological parameters, including tumor volume (Fig. 1F), lymph node metastasis (Fig. 1G), and clinical staging of patients (Fig. 1H). Notably, patients exhibiting low GATA6 expression levels in NSCLC tissues displayed a considerably poorer overall survival (Fig. 1I) and disease-free survival (Fig. 1J). Furthermore, our multivariate survival analysis showed that GATA6 was an independent prognostic factor in patients with NSCLC (*n* = 101, Table 2). Additionally, statistical analyses conducted using the KMPLOT database corroborated the prognostic significance of GATA6 mRNA expression in lung cancer patients (Fig. 1K, L). Meanwhile, we harnessed data from the TCGA and KMPLOT databases to scrutinize the expression profiles of other GATA family members in lung adenocarcinoma tissues (Fig. 2A) and their associations with the prognosis of lung cancer patients (Fig. 2B). Among the six family members, GATA6 exhibited the strongest correlation with the prognosis of lung cancer patients, implying a more pronounced involvement of GATA6 in the context of lung cancer.

**Table 1:** Correlations between GATA6 expression and clinicopathological variables

Variables	*n*	*n*		χ^2^	*p*
		GATA6 lower	GATA6 higher		
Age(years)					
< 60	74	41	33	0.439	0.507
≥ 60	76	38	38		
Gender					
Male	89	43	46	0.01	0.94
Female	61	36	25		
Smoking					
No	90	46	44	0.218	0.640
Yes	60	33	27		
T					
T1/T2	99	47	52	3.149	0.076
T3/T4	51	20	19		
N					
N0	103	46	54	8.453	0.004
N1/N2	47	33	14		
M					
M0	139	72	67	0.573	0.449
M1	11	7	4		
Clinical stage					
I–II	105	46	59	11.014	0.001
III–IV	45	33	12

**Table 2:** Multivariate analysis of survival in all population

Variables	Exp(*B*)	95.0% CI for Exp(*B*)		*p*
		Lower	Upper	
Age, years (< 60 vs. ≥ 60)	2.945	1.357	6.392	0.006
Gender (male vs. female)	0.505	0.195	1.304	0.158
T	1.113	0.519	2.389	0.783
N	3.227	1.122	9.284	0.030
M	2.503	0.526	11.904	0.249
Clinical stage	1.208	0.395	3.693	0.740
Smoking status	1.809	0.736	4.446	0.196
GATA6	0.328	0.140	0.765	0.010

### GATA6 inhibits the proliferation and migration of lung cancer cells

**Fig. 3:**
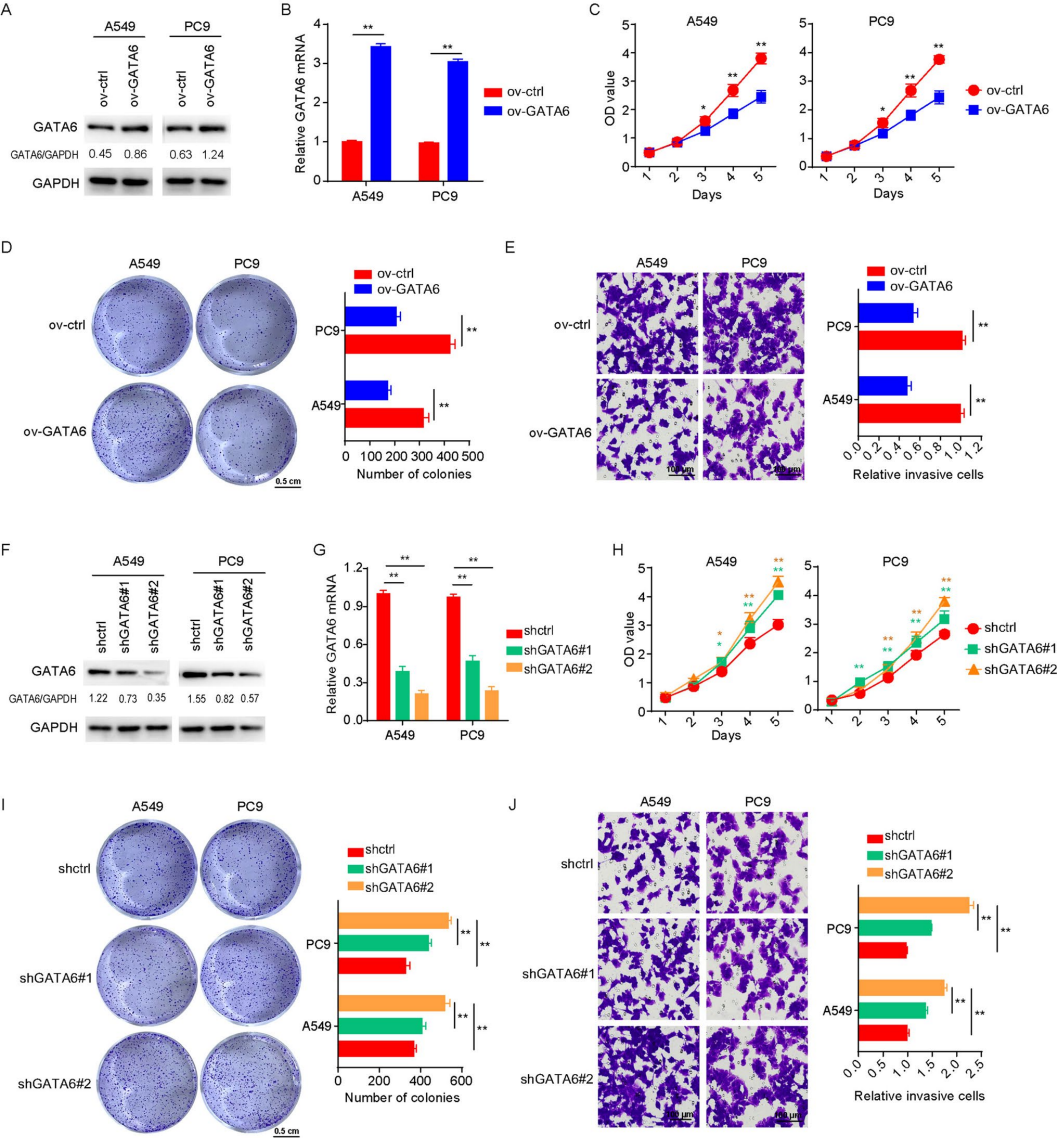
The expression level of GATA6 affects the proliferation and migration of lung cancer cells. A-B, GATA6 protein expression levels (**A**) and mRNA expression levels (**B**) in lung cancer cells A549 and PC9 cells, along with their respective GATA6 overexpression. **C**–**E**, Effect of overexpression of GATA6 on proliferation (**C**), clonal formation (**D**) and migration (**E**) of A549 and PC9. **F**–**G** GATA6 protein expression levels (**F**) and mRNA expression levels (**G**) in A549 and PC9 cells and their down-expression GATA6 cells. **H**–**J** Effect of decreased expression of GATA6 on proliferation (**H**), clonal formation (**I**) and migration (**J**) of lung cancer cells A549 and PC9

To ascertain the functional role of GATA6 in lung cancer cells, we generated cell lines with GATA6 overexpression (Fig. 3A, B), and our findings revealed a substantial inhibition of both proliferative capacity (Fig. 3C) and clonogenic potential (Fig. 3D). Moreover, the overexpression of GATA6 effectively restrained the migratory capabilities of lung cancer cells (Fig. 3E). Conversely, when GATA6 was silenced (Fig. 3F, G), it resulted in increased proliferation (Fig. 3H), heightened clonogenicity (Fig. 3I), and enhanced migration abilities (Fig. 3J) in A549 and PC9 cells. These results collectively underscore the suppressive role of GATA6 in regulating the malignant biological behavior of lung cancer cells.

### GATA6 affects the expression of glucose-related metabolic proteins

**Fig. 4:**
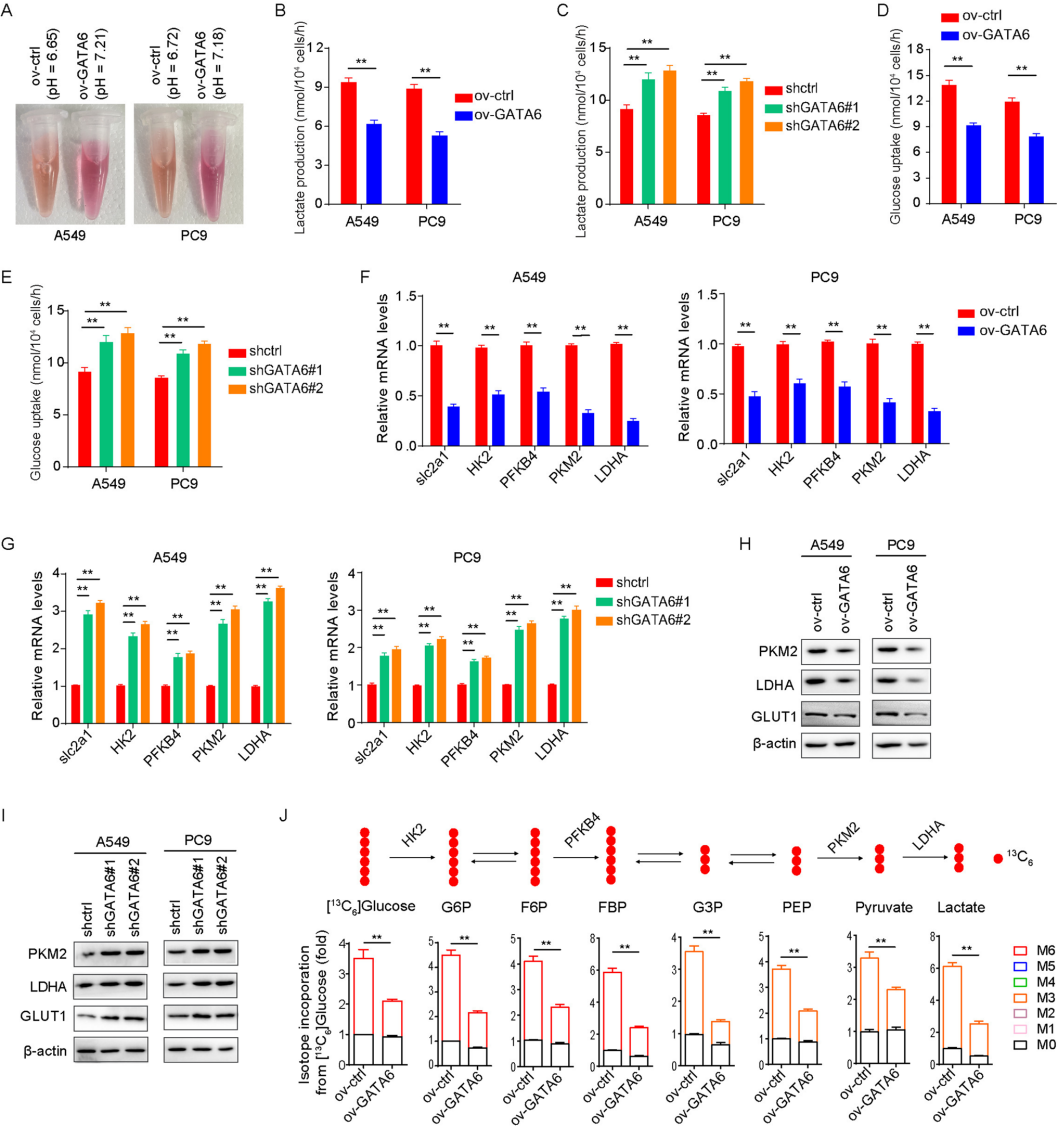
Effect of GATA6 expression level on metabolic phenotype of lung cancer cells. **A** pH value of the medium overexpressing GATA6 and its control cells. **B** Effect of overexpression of GATA6 on lactic acid production in lung cancer cells. **C** Effect of decreased expression of GATA6 on lactic acid production in lung cancer cells. **D** Effects of overexpression of GATA6 on glucose absorption in lung cancer cells. **E** Effect of decreased expression of GATA6 on glucose absorption in lung cancer cells. **F** Regulation of mRNA expression of glucose metabolism-related genes by overexpression of GATA6 in lung cancer cells. **G** Regulation of mRNA expression of glucose metabolism-related genes in lung cancer cells by decreased expression of GATA6. **H** Regulation of glucose metabolism-related protein expression by overexpression of GATA6 in lung cancer cells. **I** Regulation of glucose metabolism-related protein expression by decreased expression of GATA6 in lung cancer cells. **J** Regulation of glucose metabolism in lung cancer cell A549 by overexpression of GATA6

**Fig. 5:**
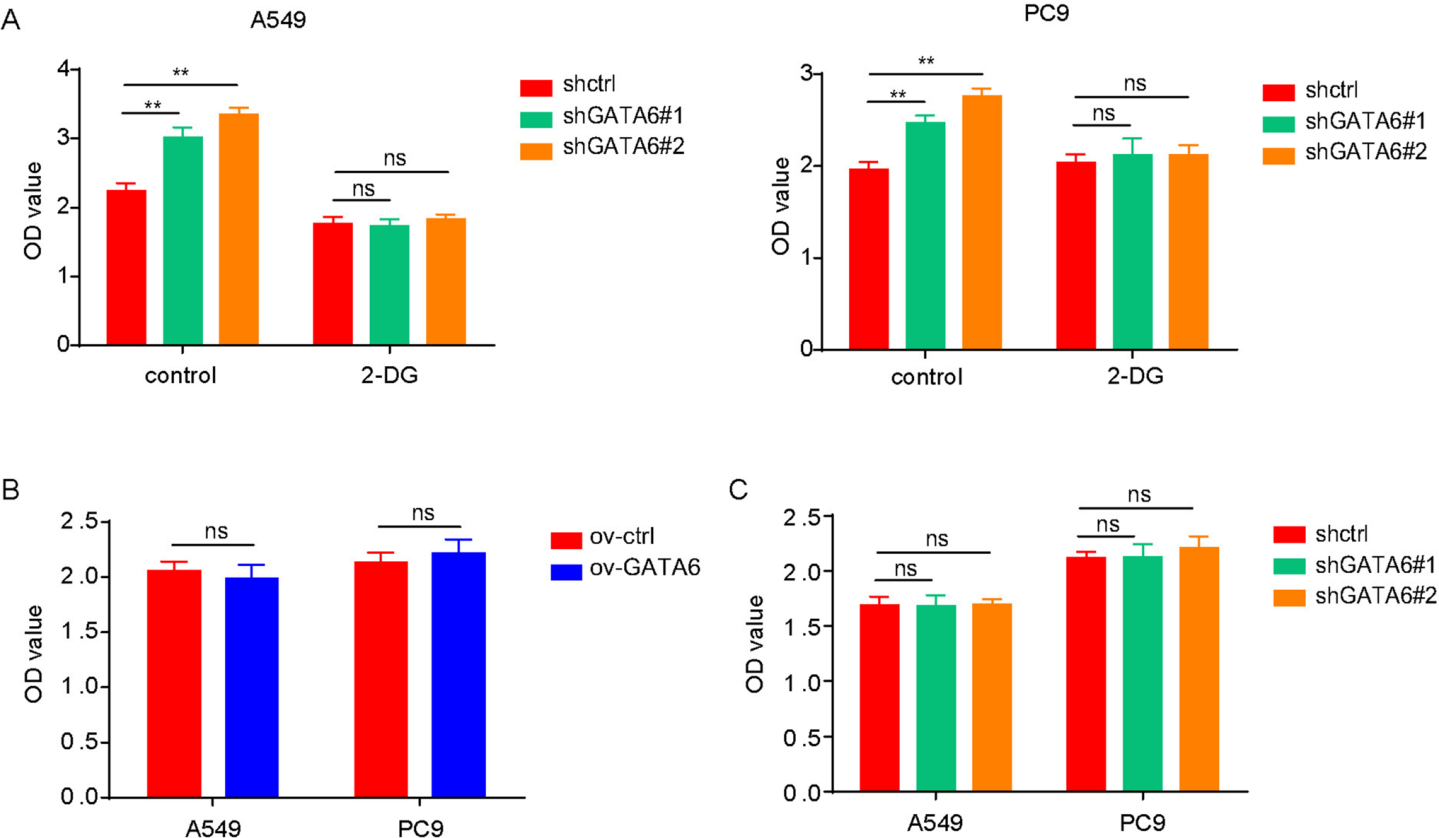
Effect of the expression level of GATA6 on the activity of lung cancer cells in sugar-free culture environment. **A** Effect of 2-DG on the viability of GATA6-expressing lung cancer cells and control cells. **B** Viability detection of lung cancer cells and control cells overexpressing GATA6 in sugar-free medium. **C** Viability detection of lung cancer cells and control cells with reduced GATA6 expression in sugar-free medium

During the cell culture process, noteworthy alterations were observed when comparing cells overexpressing GATA6 to control cells. Specifically, the medium pH of GATA6-overexpressing cells was found to be elevated (Fig. 4A), while the level of lactic acid within the medium was reduced (Fig. 4B). Conversely, GATA6-expressing cells exhibited an increase in the secretion of lactate (Fig. 4C). Additionally, GATA6 expression levels were found to be intricately linked to glucose absorption and utilization. The overexpression of GATA6 hindered the uptake and utilization of glucose by lung cancer cells (Fig. 4D), whereas decreased GATA6 expression promoted glucose uptake and utilization (Fig. 4E). Given the alterations in the metabolic phenotype of lung cancer cells, we sought to determine whether GATA6 expression levels influenced the expression of glucose-related metabolic enzymes. The results revealed that GATA6 overexpression suppressed the expression of several glucose-metabolizing enzymes as well as the glucose transporter GLUT1 (Fig. 4F, H), whereas decreased GATA6 expression fostered the expression of these proteins (Fig. 4G, I). Consequently, isotope experiments illustrated that GATA6 overexpression decelerated the metabolic flux of glucose (Fig. 4J). These findings collectively indicate that GATA6 modulates the metabolic phenotype of lung cancer cells by exerting influence over the expression of glucose-related metabolic proteins. Furthermore, the addition of 2-DG to cells with reduced GATA6 expression inhibited glucose metabolism and partially mitigated the increase in cell proliferation induced by decreased GATA6 expression (Fig. 5A). Moreover, GATA6 exhibited no significant impact on lung cancer cell proliferation in a sugar-free medium (Fig. 5B,C), underscoring its primary role in regulating glucose metabolism.

### GATA6 affects the glucose metabolism by regulating the expression of c-Myc

**Fig. 6:**
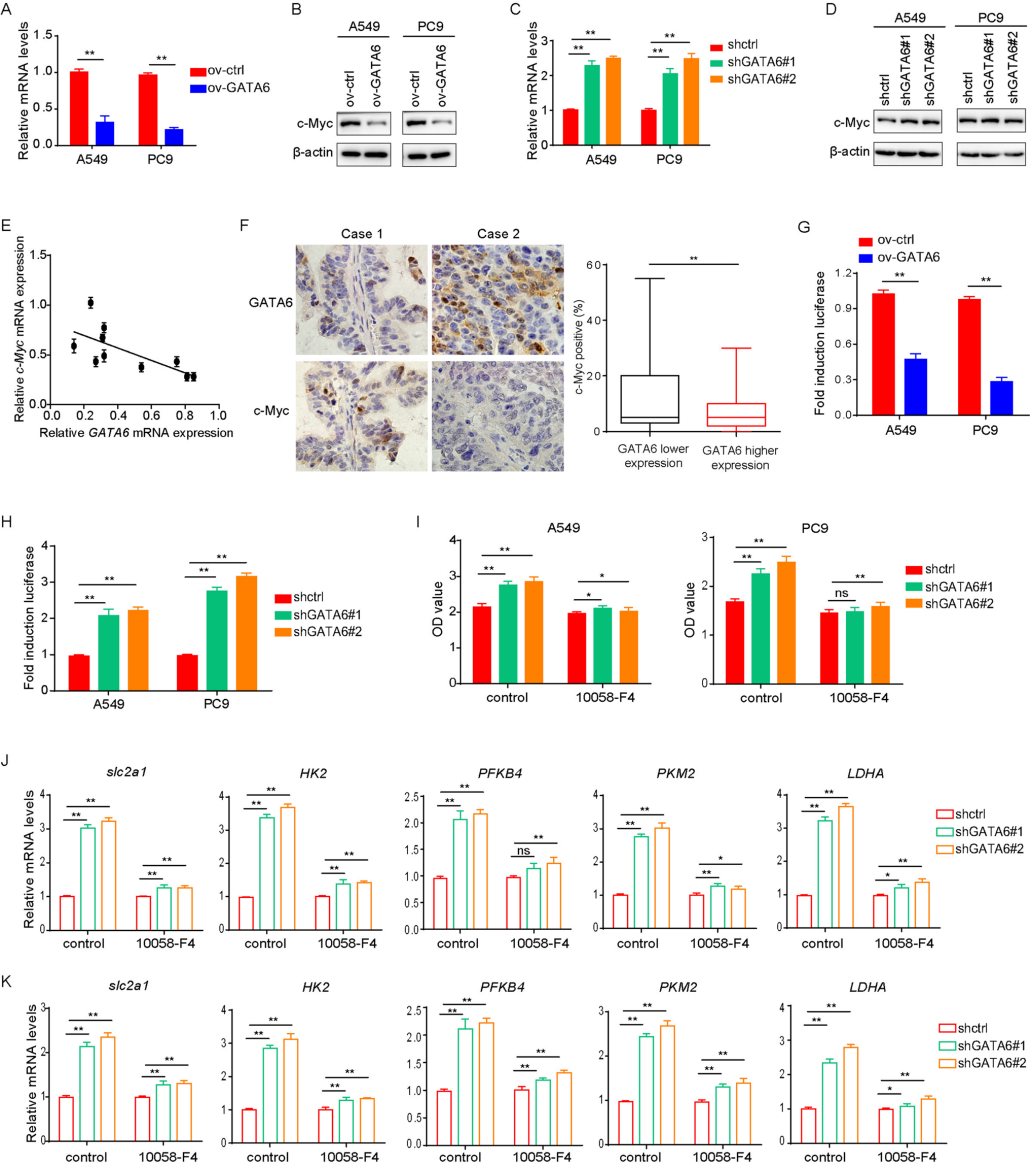
GATA6 affects the expression of glucose metabolism-related proteins by regulating the expression of c-MYC. **A** Effect of overexpression of GATA6 on expression of c-MYC mNRA in lung cancer cells. **B** Effect of overexpression of GATA6 on c-MYC protein expression in lung cancer cells. **C** Effect of decreased expression of GATA6 on c-MYC mRNA expression in lung cancer cells. **D** Effect of decreased expression of GATA6 on c-MYC protein expression in lung cancer cells. **E** Correlation between GATA6 mRNA and c-MYC mRNA expression in lung cancer samples. **F** Correlation between GATA6 and c-MYC protein expression in lung cancer samples. **G** Effect of overexpression of GATA6 on c-MYC luciferase activity in lung cancer cells. **H** Effect of decreased expression of GATA6 on c-MYC luciferase activity in lung cancer cells. **I** Effect of c-MYC inhibitor 10085-F4 on proliferation of cells with decreased expression of GATA6. **J**, **K** Effect of c-MYC inhibitor 10085-F4 on mRNA expression of glucose metabolism-related genes in A549 (**J**) and PC9 (**K**) cells with reduced GATA6 expression

C-Myc is a major transcription factor that regulates the expression of metabolic enzymes related to glucose metabolism (Fatma et al. 2022; Duffy et al. 2021; Panda et al. 2020). We aimed to investigate whether GATA6 could impact the expression of relevant metabolic enzymes by modulating c-Myc expression. Initially, our findings revealed that GATA6 overexpression suppressed the expression of c-Myc mRNA in lung cancer cells (Fig. 6A, B), whereas GATA6 underexpression produced the opposite effect (Fig. 6C, D). Furthermore, a negative correlation between GATA6 mRNA and C-MYC mRNA levels was discerned in lung cancer tissue samples (Fig. 6E). The results of IHC also corroborated these findings, indicating lower c-Myc protein expression in lung cancer tissues exhibiting higher GATA6 levels (Fig. 6F). Subsequent luciferase reporter assays provided evidence that GATA6 indeed regulated c-Myc expression (Fig. 6G, H). Moreover, the utilization of a c-Myc inhibitor mitigated the proliferation of lung cancer cells induced by GATA6 silencing (Fig. 6I), concurrently inhibiting the upregulation of mRNA expression of genes associated with glucose metabolism induced by GATA6 silencing (Fig. 6J, k). These results collectively underscore that GATA6 modulates the expression of genes implicated in glucose metabolism by regulating c-Myc expression, thereby exerting regulatory control over the functionality of lung cancer cells.

## Discussion

The GATA family of zinc finger DNA-binding proteins is indispensable for epithelial growth and the development of various tissues (Moriguchi 2021). Recent investigations have underscored the pivotal roles played by GATA family factors in tumorigenesis, including breast cancer (Song et al. 2015; Yang and Chen 2023), ovarian cancer (Shen et al. 2019; Xu et al. 2021; Gao et al. 2023), and gastric cancer (Jin and Jiang 2023). In our study, we observed a down-regulation of GATA6 expression in lung cancer tissues, and demonstrated its influence on the expression of genes implicated in glucose metabolism by inhibiting C-MYC mRNA. Consequently, this inhibition curtailed glucose uptake and metabolism by lung cancer cells, ultimately impeding their malignant biological behaviors. Our findings offer a comprehensive mechanistic insight into how GATA6 regulates the functionality of lung cancer cells, particularly from a metabolic perspective.

Currently, investigations into the relationship between GATA6 protein expression and NSCLC development have yielded inconsistent results. Chen et al. identified GATA6 as an effective lung cancer suppressor gene (Chen et al. 2020). Specifically, GATA6 inhibited AKT activation by upregulating the expression of p53 and p21 mRNA, leading to p21 protein stabilization and the induction of senescence in lung cancer cells (Chen et al. 2020). Moreover, GATA6, in synergy with HOPX, regulated overlapping alveolar differentiation and the expression of aggressive target genes, collectively limiting the metastatic potential of lung cancer cells (Cheung et al. 2013). GATA6 was also implicated in the regulation of the chromatin landscape in lung cancer cells, thereby governing tumor proliferation (Arnal-Estape et al. 2020). In addition, studies have found that miR-196b can promote the progression of lung cancer by inhibiting the expression of GATA6 (Liang et al. 2020). Conversely, other studies have suggested that GATA6 binds to the TSPAN8 promoter, promoting TSPAN8 expression, which in turn stimulated cell colony formation, proliferation, invasion, and activated the ERK signaling pathway (Xu et al. 2020). In summary, GATA6 may modulate tumor progression through diverse molecular and biochemical mechanisms, influenced by genetic backgrounds or environmental factors, resulting in either promotion or inhibition.

In our investigation, we initiated by analyzing the correlation between GATA6 expression levels and clinical parameters in lung cancer samples, revealing that both mRNA and protein levels of GATA6 were diminished in lung adenocarcinoma tissues relative to normal lung tissues. Importantly, GATA6 expression exhibited a significant negative correlation with tumor volume, lymph node metastasis, and clinical staging of patients. Lung cancer patients with reduced GATA6 expression experienced poorer overall survival and disease-free survival. These findings suggest that GATA6 may function as a tumor suppressor gene. Subsequently, our experiments unequivocally demonstrated that GATA6 overexpression significantly inhibited the proliferation and metastasis of lung cancer cells, both in vitro and in vivo.

So, what mechanism underlies GATA6's regulation of the malignant behavior of lung cancer cells? During our cell culture experiments, we observed significant disparities in the pH value and lactic acid levels within the medium among cells with varying GATA6 expression levels. Notably, the overexpression of GATA6 exhibited the capacity to suppress lactic acid production in lung cancer cells. Considering that lactic acid primarily originates from glucose, we proceeded to assess glucose uptake and metabolism in cells expressing different levels of GATA6. Our findings revealed that GATA6 overexpression inhibited both glucose absorption and utilization by lung cancer cells. Subsequent experimental validation illustrated that the overexpression of GATA6 effectively downregulated the expression of several key glucose-metabolizing enzymes, including PKM2, HK2, and LDHA, as well as the glucose transporter GLUT1. These results collectively indicate that GATA6 influences the metabolic phenotype of lung cancer cells by modulating the expression of glucose-related metabolic proteins, ultimately resulting in the inhibition of lung cancer cell proliferation and metastasis.

Given that c-Myc serves as a prominent transcription factor orchestrating the expression of metabolic enzymes associated with glucose metabolism (Camarda et al. 2017), we posited that GATA6 might influence the expression of these enzymes by regulating c-Myc expression. Subsequent experimental findings indeed substantiated our hypothesis, demonstrating that GATA6 overexpression effectively suppressed c-Myc mRNA expression in lung cancer cells. Furthermore, we observed a negative correlation between the expression levels of GATA6 and c-Myc in lung cancer tissue samples. Luciferase reporter assays provided compelling evidence of GATA6's regulatory control over c-Myc expression, which could be attenuated by the utilization of c-Myc inhibitors. Nevertheless, it is noteworthy that we did not identify a GATA motif within the promoter region of c-Myc to which GATA6 might directly bind. Consequently, we posit that the regulatory impact of GATA6 on c-Myc is likely indirect, with GATA6 potentially modulating the expression of intermediary molecules that subsequently influence c-Myc expression. The specific intermediary molecules remain to be elucidated and will require further experimental validation.

In summary, our study has revealed that GATA6 exhibits decreased expression in lung cancer tissues, and its expression levels bear significance in predicting patient prognosis. Through meticulous exploration of the underlying molecular mechanisms, we have delineated that GATA6 exerts its inhibitory effect by downregulating c-Myc expression, subsequently diminishing the expression of glucose transport and metabolism-related proteins. This orchestrated cascade ultimately results in the attenuation of glucose uptake and utilization by lung cancer cells, thereby suppressing their growth and metastatic potential. Our investigation, offering fresh insights into the role of GATA6 from the standpoint of glucose metabolism, furnishes a novel theoretical and empirical foundation with potential implications for the diagnosis and treatment of lung cancer.

## Data Availability

The datasets generated during and/or analyzed during the current study are available from the corresponding author on reasonable request.
